# Endoscopic Combined Drainage of One Giant and Multiple Small Pancreatic Pseudocysts: A Case Report

**DOI:** 10.7759/cureus.60559

**Published:** 2024-05-18

**Authors:** Yanling Wei, Fang Liu, Cuihua Qi, Hao Liu, Weigang Chen

**Affiliations:** 1 Department of Gastroenterology, The First Affiliated Hospital of Shihezi University, Shihezi, CHN

**Keywords:** endoscopic retrograde pancreatography (erp), transpapillary drainage (tpd), combining drainage (cd), endoscopic ultrasound-guided transmural drainage (eus-tmd), giant pancreatic pseudocyst, pancreatic pseudocyst (ppc)

## Abstract

A pancreatic pseudocyst (PPC) is a frequent complication of pancreatitis, often stemming from alcohol, gallstones, or hyperlipidemia. Endoscopic treatment of PPC has become the mainstream treatment. A case of one giant and multiple small PPCs was observed, manifesting as repeated abdominal bloating, abdominal pain, nausea, and vomiting after meals. Initial computed tomography scans revealed the presence of multiple PPCs. Despite ineffective medical treatment, the pseudocysts progressively increased. In response, we conducted a combined endoscopic intervention, involving Hot AXIOS (Boston Scientific, Marlborough, MA) stenting through endoscopic ultrasound-guided transmural drainage (EUS-TMD) and the placement of the endoscopic nasopancreatic drainage (ENPD) mimic stent through endoscopic retrograde pancreatography (ERP). Remarkably, after nine months of postoperative follow-up, the patient had no discomfort symptoms and the cyst disappeared. We conducted a literature review on endoscopic combined drainage for PPCs, which is still controversial. Our presented case serves as a demonstration that endoscopic combined drainage can effectively and successfully manage giant and multiple PPCs.

## Introduction

Pancreatic pseudocyst (PPC) is a localized fluid collection within or adjacent to the pancreas, enclosed by a non-epithelialized wall. It is the most common pancreatic cystic lesion, representing 75% to 80% of all cystic lesions of the pancreas [1]. The treatment options for PPCs include percutaneous drainage, surgical intervention, and endoscopic drainage. Currently, endoscopic drainage is the preferred method for managing PPCs. Pseudocysts are typically singular but may also occur as multiple entities, with those exceeding 10 cm in diameter termed as giant pseudocysts [2]. Only a few cases of "giant" PPCs have been documented to date. In this report, we detail a case of one giant and multiple small PPCs successfully treated with a combined endoscopic approach involving Hot AXIOS (Boston Scientific, Marlborough, MA) stenting through endoscopic ultrasound-guided transmural drainage (EUS-TMD) and endoscopic nasopancreatic drainage (ENPD) mimic stent placement via endoscopic retrograde pancreatography (ERP).

## Case presentation

**Figure 1:**
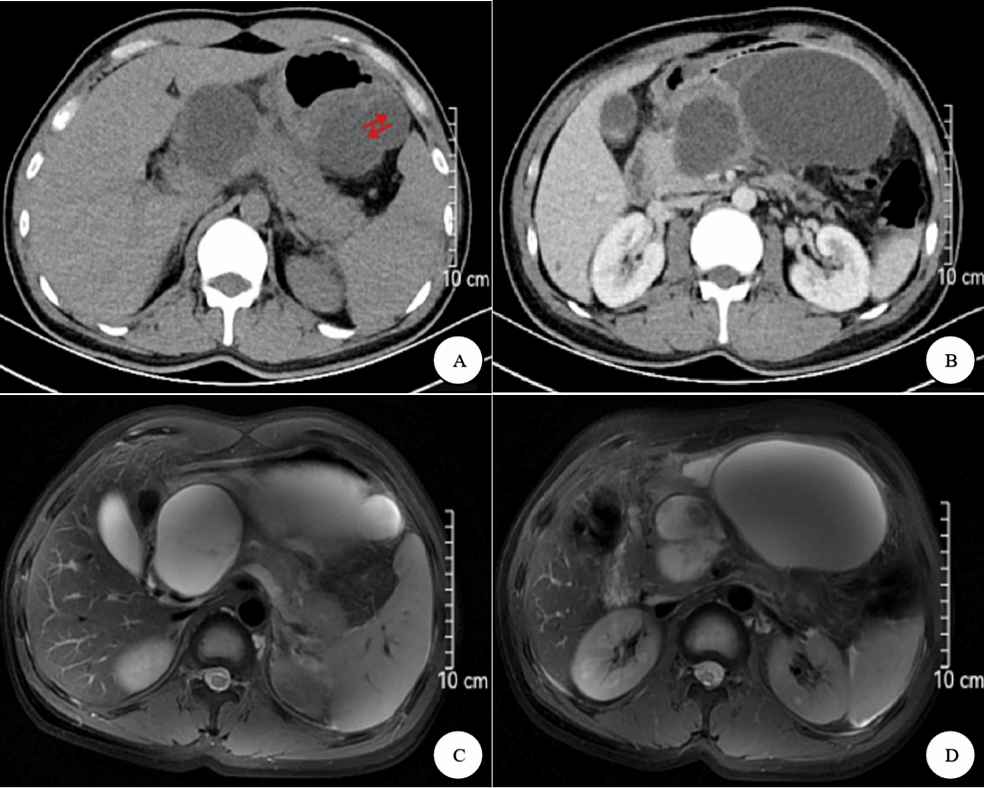
Abdominal CT and MRI, presented in coronal scans, revealed one giant and multiple small pancreatic pseudocysts distributed across the head, body, and tail of the pancreas. The largest pseudocyst, measuring approximately 122 x 79 mm, was situated in the body of the pancreas and communicated with the tail, as indicated by the red arrow.

A 36-year-old male with a BMI of 28.7 kg/m^2^ presented with recurrent abdominal pain, bloating, nausea, and vomiting, having a history of acute pancreatitis unrelated to alcohol consumption about two months ago. Physical examination revealed a large, tender upper abdominal mass, and laboratory tests showed elevated serum amylase (582 U/L). CT and MRI scans demonstrated multiple sizable PPCs throughout the pancreas, including a large one (122 x 79 mm) communicating with the tail, as illustrated in Figure 1, which was identified as a PPC.

**Figure 2:**
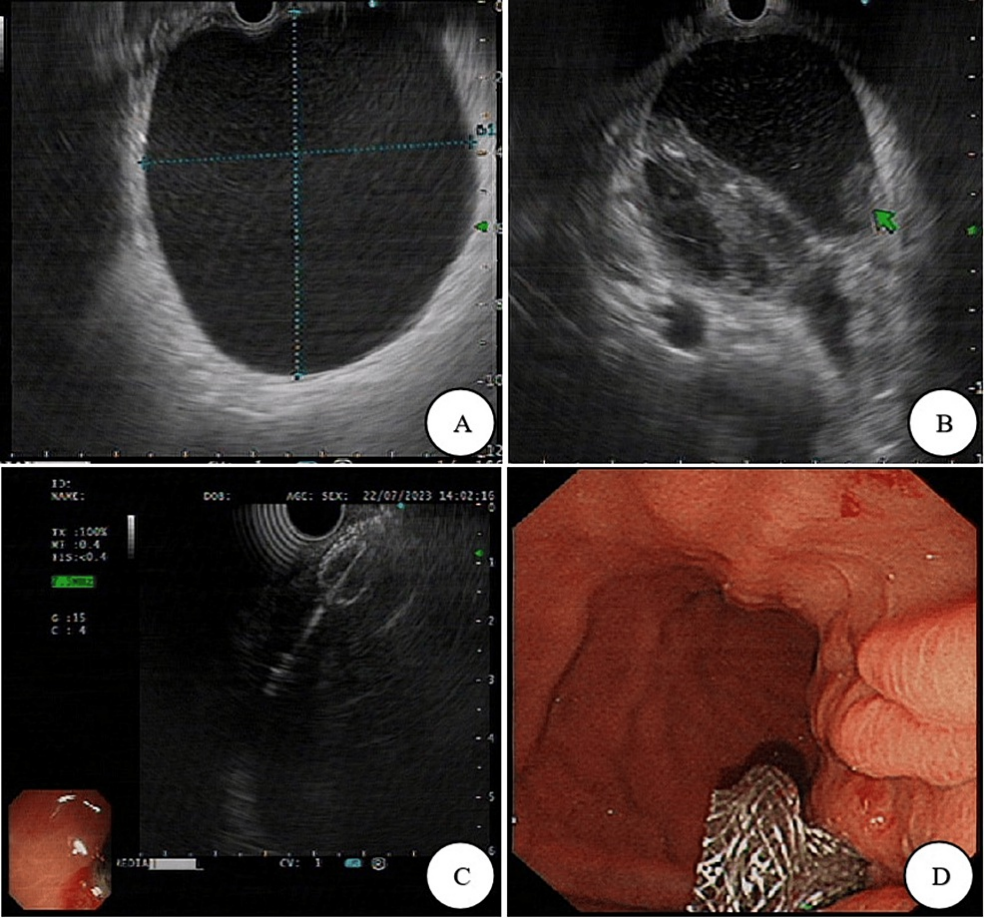
Hot AXIOS stenting through endoscopic ultrasound-guided transmural drainage (EUS-TMD) was illustrated in the following images: (A) an EUS image focusing on the body of the PPC; (B) an EUS image highlighting the head of the PPC; (C) an EUS depiction demonstrating the placement of one end of the Hot AXIOS stent into the cyst prior to puncture; (D) an endoscopic image capturing the Hot AXIOS stent within the stomach. PPC, pancreatic pseudocyst; EUS, endoscopic ultrasonography.

Despite the conservative medical therapy being done actively, the patient still exhibited the above symptoms and had lost a considerable 20 kg of weight, which means our drug treatment was found to be futile. After a multidisciplinary case discussion, endoscopic drainage of the PPC was recommended. EUS-TMD with a lumen-apposing metal stent (Hot AXIOS) was performed successfully, as shown in Figure 2.

**Figure 3:**
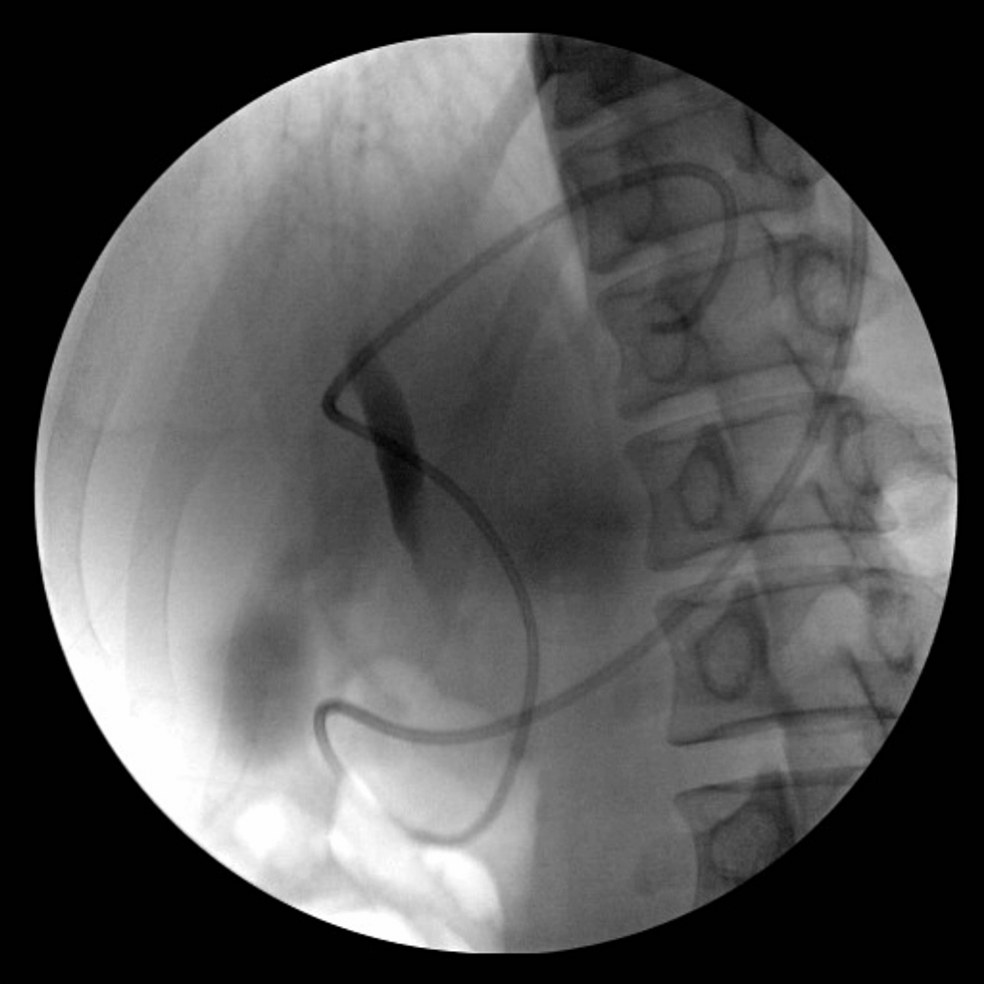
ENPD mimic stent placement by ERP. ENPD, endoscopic nasopancreatic drainage; ERP, endoscopic retrograde pancreatography.

After one week, the patient reappeared for abdominal pain after the reduction of abdominal pain. Repeat CT scans indicated a gradual reduction in the size of the pancreatic body and tail cysts, but the head cyst showed progressive enlargement to 98 x 73 mm. Moreover, both endoscopic ultrasound (EUS) and MRI revealed that the pancreatic head cyst communicated with the pancreatic duct. Consequently, ERP and ENPD were smoothly performed, as illustrated in Figure 3.

Five days later, the ENPD drainage duct became obstructed, leading to a high fever in the patient. Based on the bacterial culture and antimicrobial susceptibility of cyst fluid identifying *Klebsiella pneumoniae *(Table 1), piperacillin-tazobactam was used as anti-infective therapy, alongside other necessary treatments. Following the stabilization of the patient's condition, he was discharged from the hospital.

**Table 1:** Biochemical analysis and bacterial culture of cyst fluid. CEA, carcinoembryonic antigen; CA199, carbohydrate antigen 199; EUS-TMD, endoscopic ultrasound-guided transmural drainage; ERP, endoscopic retrograde pancreatography; ENPD, endoscopic nasopancreatic drainage.

Location	Amylase (U/L)	Glucose (mmol/L)	CA199 (U/ml)	CEA (ng/ml)	Bacterial culture	Operation
Body	71115	4.31	556.4	10.59	Staphylococcus aureus	EUS-TMD
Head	45447	3.5	>1000	0.64	Pseudomonas aeruginosa	ERP + ENPD
Head	-	-	809.1	0.9	Klebsiella pneumoniae	5 days after ERP + ENPD

**Figure 4:**
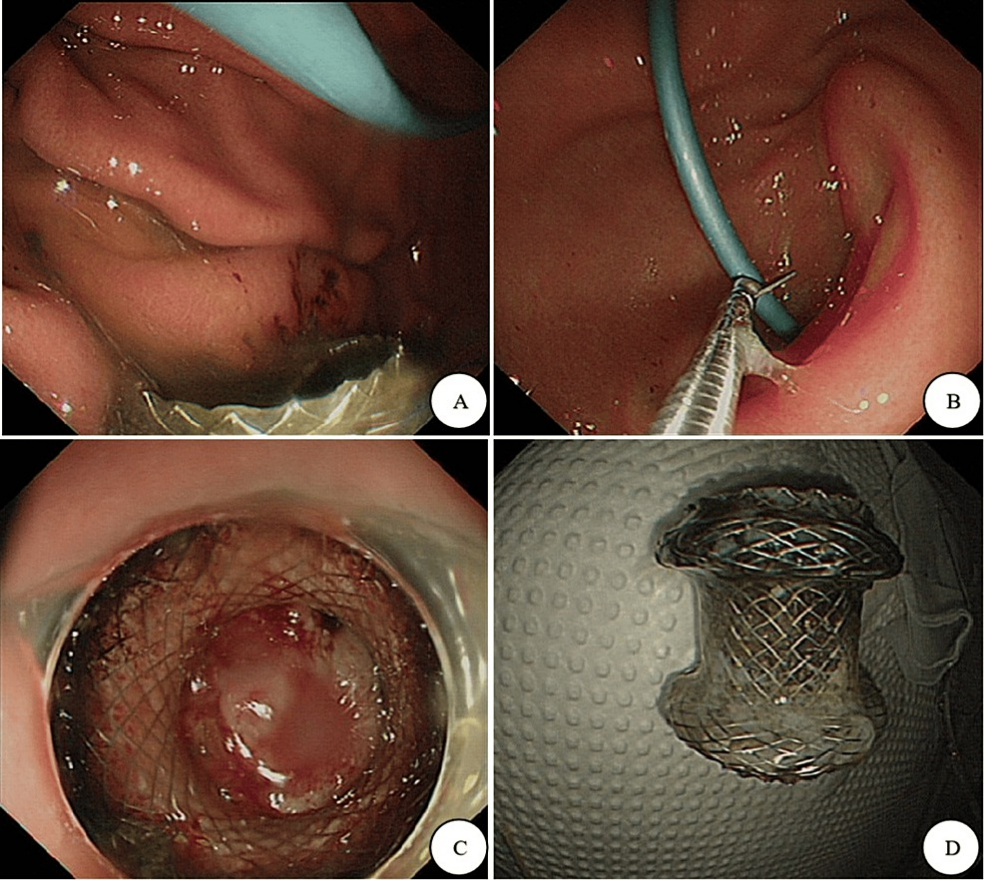
Endoscopic images at eight weeks after endoscopic ultrasound-guided transmural drainage (EUS-TMD): (A) the nasal pancreatic duct and the stent were present in the stomach; (B) the nasal pancreatic duct was severed; (C) an endoscopic image capturing the Hot AXIOS stent within the stomach; (D) the Hot AXIOS stent positioned in the stomach was removed.

Eight weeks later, the Hot AXIOS stent positioned in the stomach was removed, and the nasal pancreatic duct was severed. Subsequently, four months later, the ENPD mimic stent was also removed, as illustrated in Figure 4.

**Figure 5:**
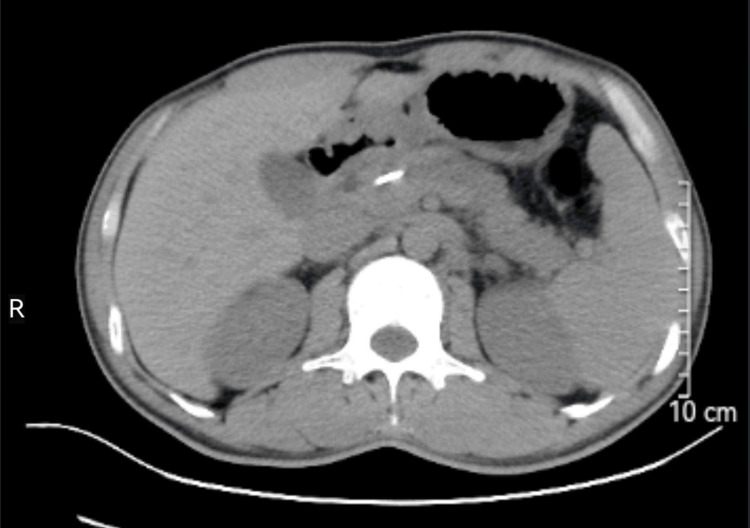
CT scan after four months post-initial procedure indicated the total disappearance of the cyst in the head and body of the pancreas.

After the ENPD mimic stent was removed, the cysts in the head and body of the pancreas disappeared totally (as illustrated in Figure 5), but then the cyst in the head reappeared again. The abdominal ultrasound examination suggested that the maximum diameter of the cyst in the head was 4 cm, which is currently 2.1 cm. Moreover, the patient has no uncomfortable symptoms and is still under follow-up.

## Discussion

PPCs commonly arise as a secondary consequence of acute pancreatitis, chronic pancreatitis, pancreatic injury, or pancreatic surgery. Although pseudocysts typically lack specific symptoms, the most prevalent manifestations include abdominal pain (76-94%) and nausea and vomiting (50%), with weight loss observed in 20-51% of cases. Over 50% of instances where acute pancreatitis is accompanied by pseudocysts remain asymptomatic and tend to resolve naturally without intervention [3]. However, when the cyst attains a significant size (>6 cm) and induces compression symptoms or complications, active intervention is warranted.

PPC treatment options, including percutaneous catheter drainage (PCD), surgical drainage (SD), and endoscopic drainage (ED), favor the latter as the optimal approach in contemporary practice. ED encompasses transmural drainage (TMD) and transpapillary drainage (TPD), with the choice dependent on cyst location, connection to the main pancreatic duct, ductal obstruction, and the physician's experience. TPD involves balloon dilation and stenting under ERP, necessitating communication between the main pancreatic duct and the pseudocysts. In contrast, TMD can be accomplished across the duodenal or gastric wall under EUS, requiring pseudocysts to be close to the gastrointestinal wall (<1 cm). Metal stents, particularly the innovative Hot AXIOS system, are preferred for EUS-TMD in treating PPCs due to their efficacy and safety, as demonstrated by Li et al. [4]. If a pseudocyst does not heal after a single drainage procedure, a combination of TPD and TMD may be considered, although the benefits of such a combined approach remain limited and controversial, as outlined in Table 2. According to a large multi-center and retrospective study, TPD has no benefit on treatment outcomes in patients undergoing EUS-guided TMD of PPCs and negatively affects the long-term resolution of PFCs [5]. Additionally, Libera et al. reported that different endoscopic drainage routes, including TMD, TPD, and CD, did not differ in the efficacy of PPCs [6], and Hookey et al. suggested a higher recurrence rate when PFCs were drained by combined transpapillary and transmural techniques compared with transmural alone [7]. On the contrary, other studies reported a major benefit of TPD on treatment outcomes of patients undergoing TMD. Studies by Trevino et al. and Shrode et al. reported that TPD would improve the treatment outcomes of TMD in cases with partial pancreatic ductal disruptions in PFCs [8,9]. Furthermore, recent research by Ni et al. indicated that transpapillary pancreatic duct stenting seems to improve the efficacy of endoscopic TMD of pancreatic duct disruption-associated PFCs by reducing the recurrence rate and shortening the length of hospital stay [10].

**Table 2:** Characteristics of studies on endoscopic combined drainage of PPC. PPC, pancreatic pseudocysts; PFC, pancreatic fluid collections; WON, walled-off necrosis; TPD, transpapillary drainage; TMD, transmural drainage; CD, combined drainage; PD, pancreatic duct; NR, no record.

Study	Type	Year	Country	Sample size (n)	Population age, year	Size of PFC (mm), mean	Type of PFC	Drainage route	Technical success rate	Complications	Recurrence rate	Median follow-up	CD benefit or not
Yang et al. [5]	Retrospective	2015	USA	375	52.7 TMD, 50.9 CD	90 TMD, 95 CD	375 PFC (174 PPC)	95 TMD, 79 CD	TMD 97%, CD 44%	TMD 14.7%, CD 13.9%	NR	TMD 324 days, CD 201 days	×
Libera et al. [6]	Prospective	2000	Brazil	30	38.4	91	30 PPC	8 TPD, 12 TMD, 5 CD	TPD 75%, TMD 83%, CD 100%	16.20%	4.8%	42 weeks	×
Hookey et al. [7]	Retrospective	2006	Belgium	116	51.2	60	116 PFC (94 PPC)	15 TPD, 60 TMD, 41 CD	87.90%	11%	TPD 20%, TMD 8.3%, CD 26.8%	21 months	×
Trevino et al. [8]	Retrospective	2010	USA	110	51.7	100.6	68 PPC, 20 abscesses, 22 necrosis	40 CD, 70 TMD	CD 97.5%, TMD 80%	3.20%	CD 7.7%, TMD 3.6%	9.9 months	√
Shrode et al. [9]	Retrospective	2013	USA	113	51.3	NR	113 PPC	36 TPD, 36 TMD, 33 CD	Partial PD disruptions: TPD 75%, TMD 100%, CD 78.5%. Complete PD disruption: TPD 75%, TMD 72.7%, CD 57%	26.50%	NR	15.3 months	√
Ni et al. [10]	Retrospective	2023	China	153	47.7	84	57 PPC, 96 WON	6 TPD, 15 TMD, 36 CD	TPD 100%, TMD 93.3%, CD 97.2%	TPD 0, TMD 6.7%, CD 5.6%	TPD 16.7%, TMD 6.7%, CD 0	12 months	√

However, the above studies on endoscopic combination therapy of pseudocyst are not suitable for giant PPCs. Until now, the management of giant PPCs lacks an established standard due to the rarity of the condition. In our case, a successful combination of TPD and TMD was performed. A CT scan during the four-month follow-up post-initial procedure revealed no evidence of fluid re-accumulation. After that, the abdominal ultrasound examination every half a month suggested that the cyst in the head of the pancreas had reappeared. Considering the cyst was small and the patient had no discomfort symptoms, it was still in follow-up. By the time of publication, the patient has been followed up for nine months. Given the complexity of giant and multiple PPCs, repeated procedures may be necessary to ensure proper drainage and necessitate close follow-up. This case underscores the efficacy of endoscopic combined drainage in successfully managing giant and multiple PPCs.

## Conclusions

A giant PPC is a rare yet potentially life-threatening complication of pancreatitis. Achieving an optimal treatment outcome requires an individualized, multidisciplinary approach, considering factors such as the number, size, and location of the PPC, its connection with the main pancreatic duct, ductal obstruction, patient symptoms, and the physician's experience. In our case, the utilization of endoscopic combined drainage, involving EUS-TMD and ENPD mimic stent placement via ERP, proved effective in decompressing giant and multiple PPCs.
